# Evaluation of Sequential Head Computed Tomography in Traumatic Brain Injuries

**DOI:** 10.7759/cureus.27772

**Published:** 2022-08-08

**Authors:** Sachin P Shetty, Anupama Chandrappa, Sudha K Das, Kamal K Sen, Divya V Kini

**Affiliations:** 1 Radiology, Jagadguru Sri Shivarathreeshwara (JSS) Medical College, Mysuru, IND; 2 Radiology, Kalinga Institute of Medical Sciences, Bhubaneswar, IND

**Keywords:** road traffic accident, head injury, radiation dose, computed tomography, rotterdam score

## Abstract

Background: The grading of the severity of head trauma plays a vital role in acute patient management and planning a case-appropriate follow-up protocol. Few studies have been published regarding the Rotterdam scoring. In this study, we have established a correlation between the Rotterdam scores, need for sequential CTs, and the cumulative radiation dose. This correlation has helped develop a preliminary protocol that can be followed for patients hence bringing about better planned and efficient patient care.

Materials and methods: From August 2014 to December 2020, 88 cases of traumatic head injury on whom a minimum of one sequential CT was performed, with no surgical intervention, were included and studied. Sequential head CTs of each patient were evaluated by skilled radiologists with a minimum experience of five years, all of whom were blinded to the findings of the initial and previous head CT findings. The serial head CTs were evaluated for the Rotterdam CT score (RCTS).

Results: Among the patients with extradural hemorrhage (EDH), only 28.6% (8) progressed over successive CTs and 75.5% (34) of patients with subdural hemorrhage (SDH) showed significant progression over sequential CTs. Maximum number of serial CTs were obtained for cases presenting with a score of 3 (34 cases) with about three of them requiring up to a total of three CTs. However, no significant change in findings was noted on serial CTs. On the contrary, significant disease progression was noted in patients with baseline scores of 4 (76.9%) and 5 (100%), with statistical significance obtained on further analysis (P = 0.001).

Conclusions: We are of the opinion that there is no additional role of sequential CT for the cases with Rotterdam score of 1 or 2 in the initial CT unless there is clinical evidence of deterioration. Rotterdam score 3 needs sequential CT after 24 hours and Rotterdam scores 4 and 5 need sequential CT after 12 hours if surgical intervention is delayed. The Rotterdam score may help predict any further need for a second CT, hence decreasing the unwanted radiation exposure.

## Introduction

According to Hippocrates, "no head injury is so minor as to be neglected, nor so serious as to be disappointed." In rapidly developing countries, high rates of development and industrialization have led to multifold growth in the number of road transportation and with resultant steady increase in road traffic accidents (RTA). Most victims are young, healthy members of the working population who are vital to the economy in addition to emotional burden of suffering for themselves and their families [1]. A paradigm shift over the last decade is perceptible; a sense of urgency for prompt diagnosis and neuroimaging assessment of trauma is visible. Neurotrauma in the current scenario is not only identified, but evaluated and quantified. Previously, the mainstay of diagnosis of intracranial traumatic lesions was at best clinical evaluation, plain roentgenograms of skull, and cerebral angiography. The advent of CT and the recent influx of newer generations of multidetector computed tomography (MDCT) has revolutionized the understanding of traumatic brain injury (TBI) [2].

Head CT has eased diagnosis and paved the way for classification of TBIs based on etiology, pattern of injury in correlation with pathoanatomical distribution, and CT scoring systems viz, Marshall CT score and Rotterdam CT score that have aided in prognosticating outcomes in neurotrauma. The primary goal in treating patients with craniocerebral trauma due to any cause is to preserve the patient's life and remaining neurological function. Optimal management of these patients depends on early and correct diagnosis and therefore neuroimaging has a vital role [2].

In this study, we have assessed the role of serial head CT scans in patients with TBI and correlated the pattern of traumatic brain injury and its evolution on serial CT scans. Additionally, the cumulative radiation dose was quantified in such patients who were subjected to repeat CT head during their course in this hospital.

## Materials and methods

A prospective observational study was performed on over 88 patients at the department of radio-diagnosis in our institution for all patients with traumatic brain injury who underwent sequential head CT during the period from August 2014 to December 2020 and who fulfilled our inclusion criteria.

Head CT of each patient was evaluated by skilled radiologists with a minimum experience of five years, all of whom were blinded to the findings of the initial and or previous head CT findings. The serial head CTs were evaluated with the Rotterdam CT score (RCTS) for potentially deteriorating lesions on the initial CT scan that may lead to progressive hemorrhagic injury. Worsening RCTS were correlated to modification of therapy.

Rotterdam CT score and the lesion that could lead to secondary hemorrhagic injury were also documented (Table 1). Calvarial fractures associated with extradural and subdural hematoma were also documented. Cumulative radiation dose as a net result of all sequential CTs was calculated for each patient.

**Table 1:** Rotterdam scoring for assessment of severity of TBI on head CT SAH: subarachnoid hemorrhage; TBI: traumatic brain injury

Basal cisterns	0	Normal	The final score is the sum of the scoring items + 1.
	1	Compressed	
	2	Absent	
Midline shift	0	No shift or ≤ 5mm	
	1	Shift > 5mm	
Epidural mass lesion	0	Absent	
	1	Present	
Intraventricular blood or traumatic SAH	0	Absent	
	1	Present	

All patients with acute traumatic brain injury above the age of 15 years and patients who have been subjected to two or more CT scans of the brain pre-operatively were considered as part of our study.

Patients were taken up for surgery based upon the findings of the first CT scan, those who were discharged or who expired after the first CT scan of head and those whose initial CT of the head was done elsewhere were excluded from the study.

CT of the head was performed using 128 slice MDCT scanner (Philips Ingenuity; Cleveland, OH: Philips Medical Systems) with field-of-view (FOV) from the vertex of the skull to the angle of the mandible. Collimation of 64 × 0.625 with slice thickness of 3 mm and increment of 1.5 mm was set with appropriate exposure factors - 120 kVp and 350 mAs.

Statistical analysis was performed using SPSS software, version 22.0 (Armonk, NY: IBM Corp.). Descriptive statistics were used to display univariate summary statistics and calculate standardized z-values. Chi-square test was used to compare the observed and expected frequencies in each category to test that all categories contain the same proportion of values. Further, Cramer’s V (cross-tabulation) was used to form two-way and multiway tables to assess percentage data to the total. Degree of association between various parameters was assessed by Pearson’s correlation. Statistical significance was set at a two-sided P <0.05.

## Results

Of all the trauma cases referred to our institute over the last seven years, 88 cases were selected that fulfilled our inclusion criteria. Of these, 84 patients were males as compared to only four female patients. The occurrence of significant traumatic brain injury was in the age group 20-50 years (64%).

Twenty-eight patients presented with extradural hemorrhage (EDH), 45 with subdural hemorrhage (SDH), and 61 with subarachnoid hemorrhage (SAH). Sixty-one cases presenting with trauma had SAH on imaging and a total of 40 cases presented with intraparenchymal bleed.

**Figure 1:**
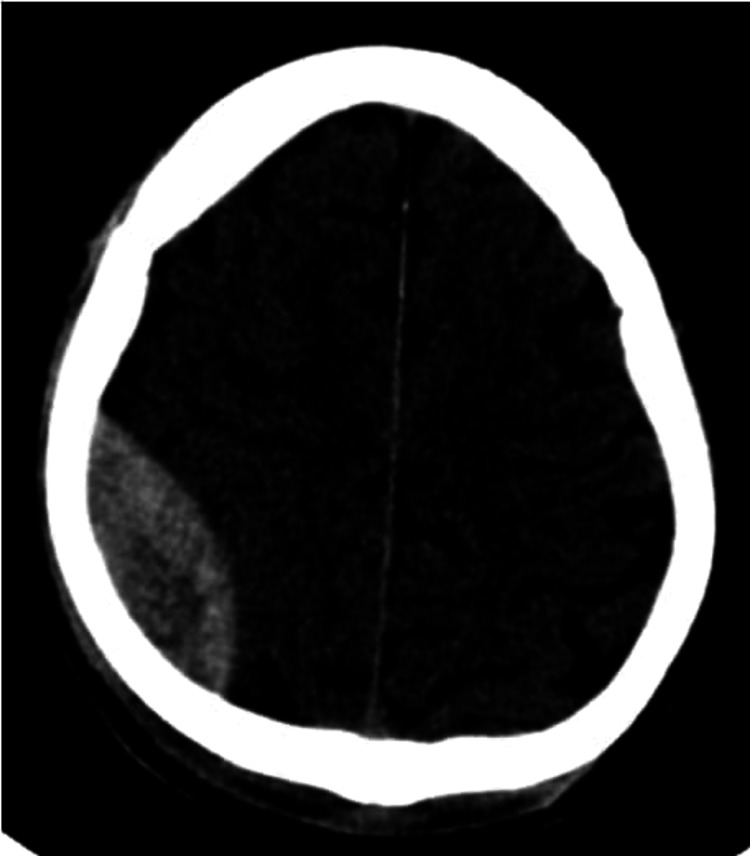
Axial NECT shows an extradural hemorrhage in the right parietal region NECT: non-contrast-enhanced computed tomography

**Figure 2:**
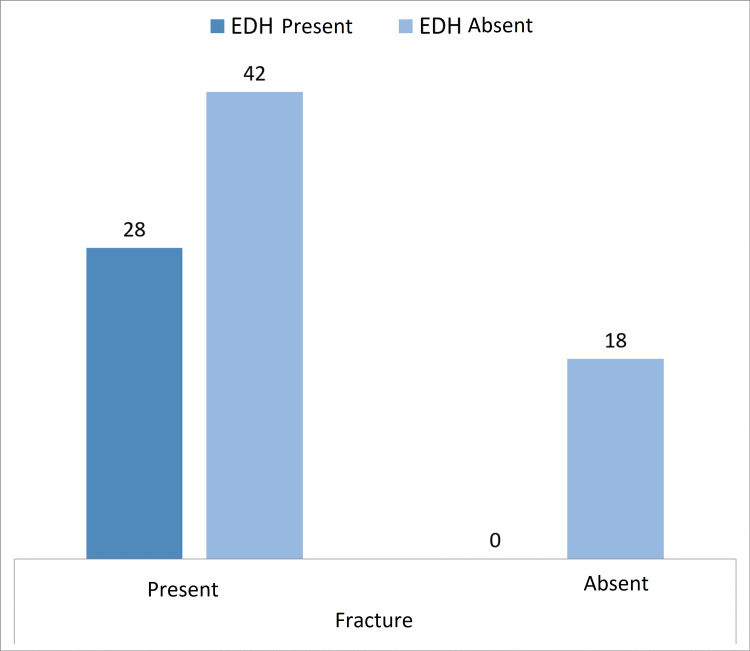
Graphical representation of the association of EDH with fractures EDH: extradural hemorrhage

Among the patients with EDH (Figure 1), all of the 28 cases had associated skull fractures (Figure 2). Only eight (28.6%) of these progressed over successive CTs resulting in increased RT scores and warranting repeat CTs. On the other hand, 20 cases (71.4%) did not show any evidence of progression on repeat CTs (Table 2). Of these cases, 53.6% developed midline shift secondary to the mass effect (Table 3).

**Table 2:** Cross-tabulation of EDH with progression of EDH EDH: extradural hemorrhage

			EDH progression		
			Absent		Present
EDH	Absent	N (%)	60 (100%)		0.00%
						Present	N (%)	20 (71.4%)		8 (28.6%)
	Total		N (%)	80 (90.9%)							8 (9.1%)

**Table 3:** Cross-tabulation of EDH with midline shift EDH: extradural hemorrhage

			Midline shift	
			Absent	Present
EDH	Absent	N (%)	46 (76.7%)	14 (23.3%)
					Present	N (%)	13 (46.4%)	15 (53.6%)
	Total		N (%)	59 (67%)					29 (33%)

**Figure 3:**
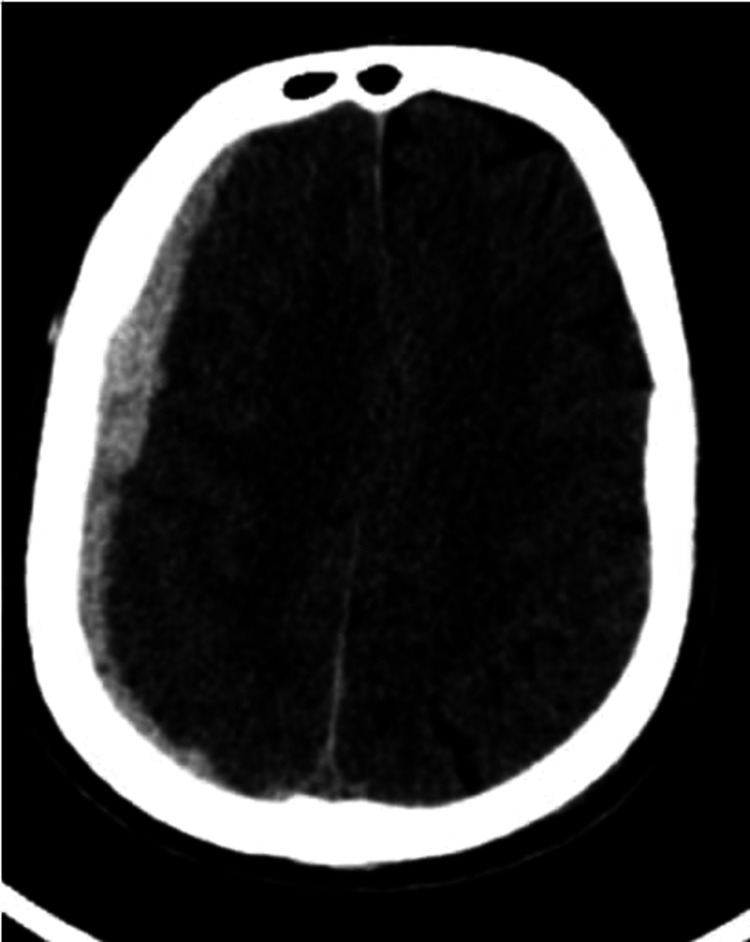
Axial NECT shows a subdural hemorrhage in the right cerebral hemisphere NECT: non-contrast-enhanced computed tomography

**Figure 4:**
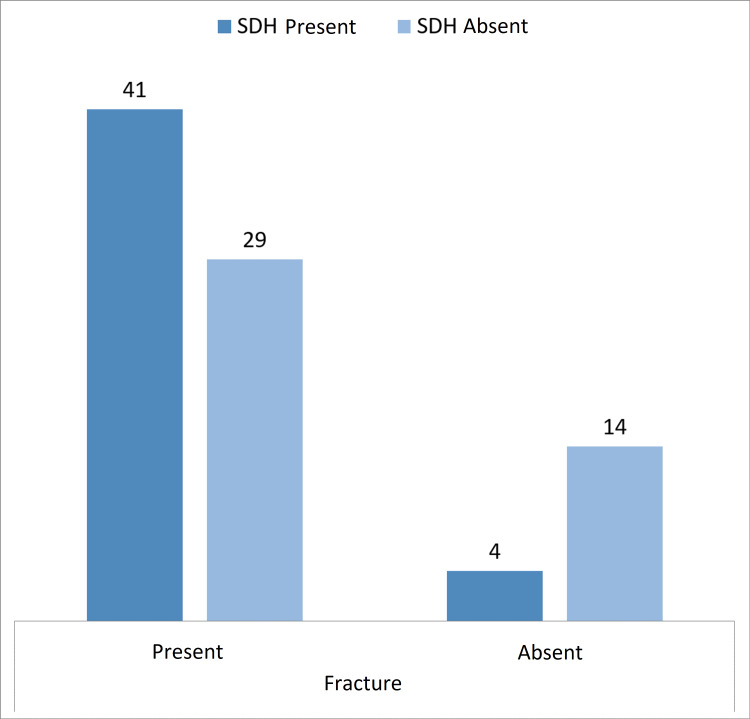
Graphical representation of the association of SDH with fractures SDH: subdural hemorrhage

**Figure 5:**
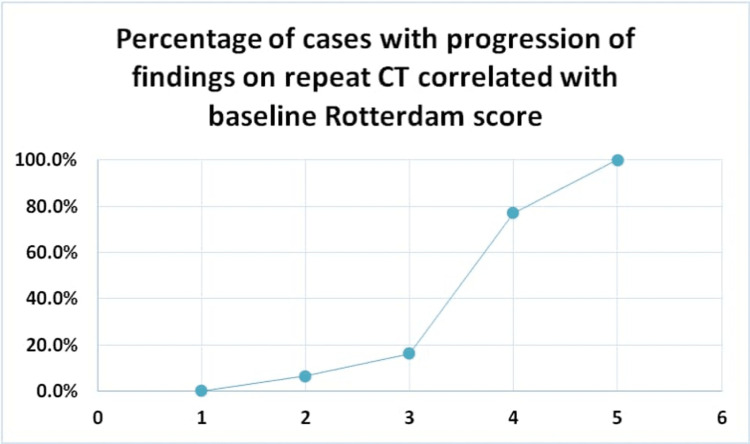
Graphical representation of the percentage of cases with progression of findings on repeat CT correlated with baseline Rotterdam score

Forty-five patients presented with SDH (Figure 3) of which 41 (91.1%) cases were associated with fractures (Figure 4). Of these cases, 75.5% (34) showed significant progression over sequential CTs (Figure 5 and Table 4) and 40% of the cases developed midline shift while 60% of the cases remained stable over repeat CT scans (Table 5).

**Table 4:** Cross-tabulation of SDH with progression of SDH SDH: subdural hemorrhage

			SDH progression	
			Absent	Present
SDH	Absent	N (%)	43 (100%)	0 (0%)
					Present	N (%)	34 (75.5%)	11 (24.5%)
	Total		N (%)	77 (87.5)%					11 (12.5%)

**Table 5:** Cross-tabulation of SDH with midline shift SDH: subdural hemorrhage

			Midline shift	
			Absent	Present
SDH	Absent	N (%)	32 (74.4%)	11 (25.6%)
					Present	N (%)	27 (60%)	18 (40%)
	Total		N (%)	59 (67%)					29 (33%)

Evidence of progression on sequential CTs was noted in a total of 33 cases (37.5%), of which, 23 cases required treatment modification while the remaining 10 cases were continued on the same management protocols. Fifty-one of these cases did not show progression on subsequent CTs and neither required any form of treatment modification (Table 6).

**Table 6:** Tabular representation of progression of imaging findings on sequential CTs and correlation with treatment modification

		Rotterdam score progression	
		Yes	No
Treatment modification	Yes	23	4
	No	10	51

**Figure 6:**
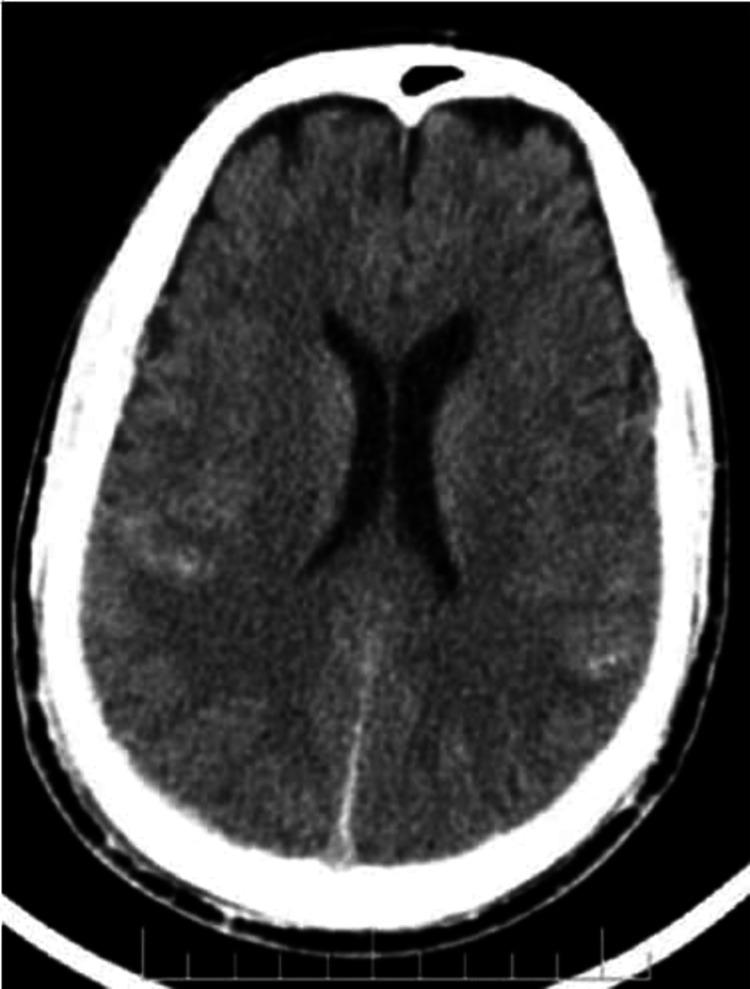
Axial NECT shows a subarachnoid hemorrhage in bilateral parietal regions with no mass effect or compression of basal cisterns - Rotterdam score 2 NECT: non-contrast-enhanced computed tomography

**Figure 7:**
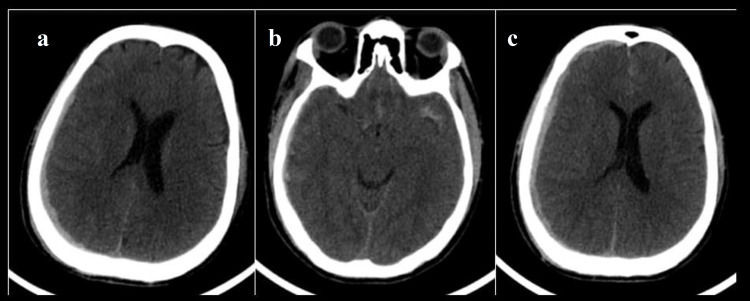
CT of a 47-year-old male patient following road traffic accident Initial admission CT shows (a) soft tissue subdural hemorrhage in the right parietal convexity causing mild effacement of the right lateral ventricle and leftward midline shift of 3 mm. Right holo-hemispheric edema was noted. Basal cisterns were normal - Rotterdam score 2. Sequential CTs after 24 hours show (b) left Sylvian cisternal subarachnoid hemorrhage and (c) increase in the thickness of subdural hemorrhage, however, midline shift did not increase. Right holo-hemispheric edema was noted. Basal cisterns were normal - Rotterdam score 3.

**Figure 8:**
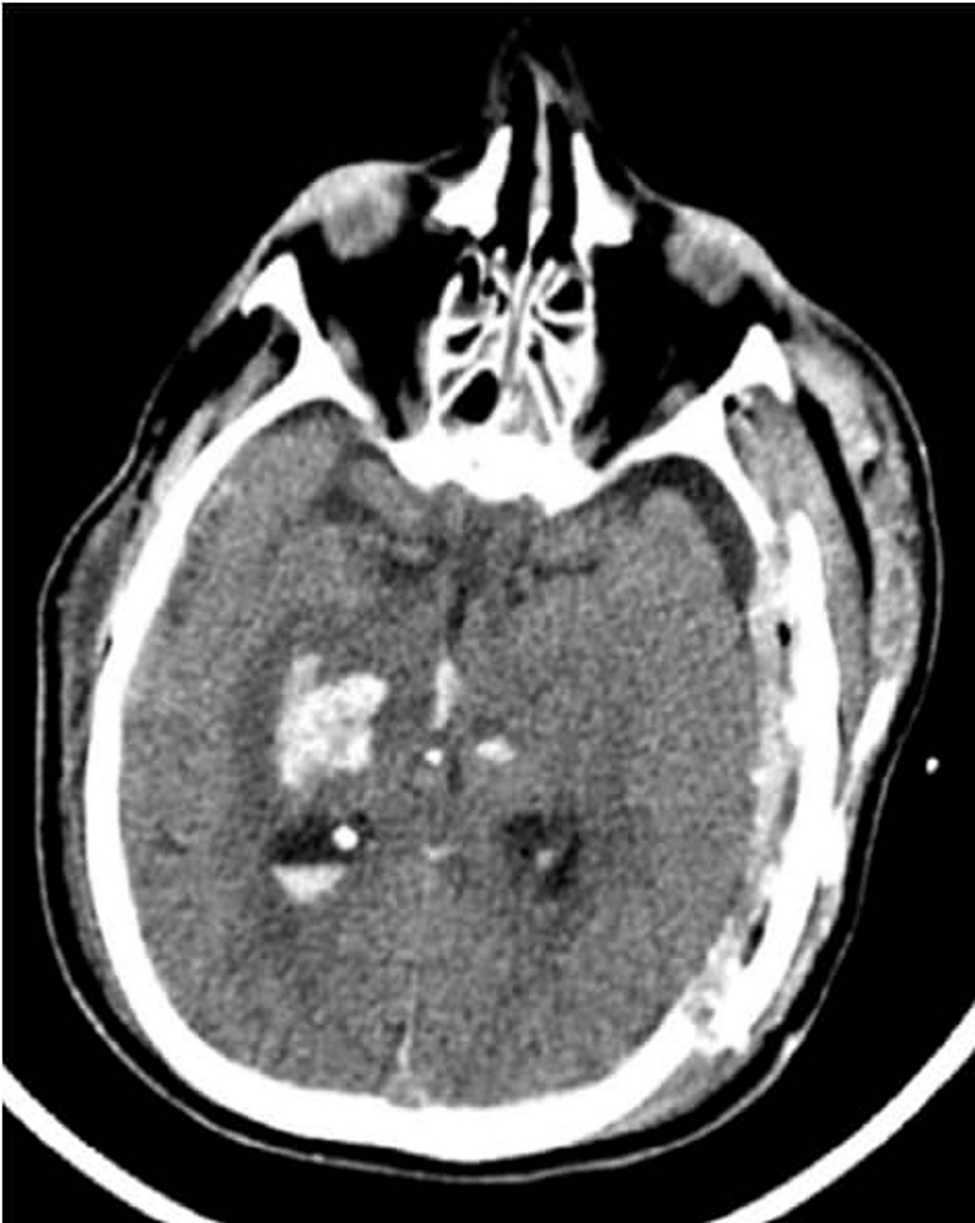
Axial NECT shows an intraparenchymal contusion in right capsulo-ganglionic region and intraventricular bleed noted with minimal mass effect compressing basal cisterns - Rotterdam score 3 NECT: non-contrast-enhanced computed tomography

**Figure 9:**
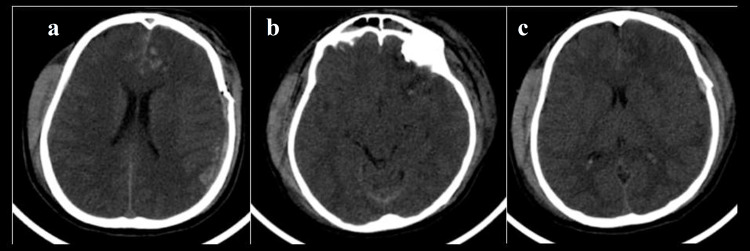
CT of a 22-year-old male patient following road traffic accident Initial admission CT shows (a) soft tissue swelling in the right parietal region with extradural hemorrhage in the left parietal convexity and traumatic subarachnoid hemorrhage in bilateral frontal lobes - Rotterdam score 3. Sequential CTs after 24 hours show (b) effacement of basal cisterns and (c) cerebral sulcal spaces - Rotterdam score 4.

**Figure 10:**
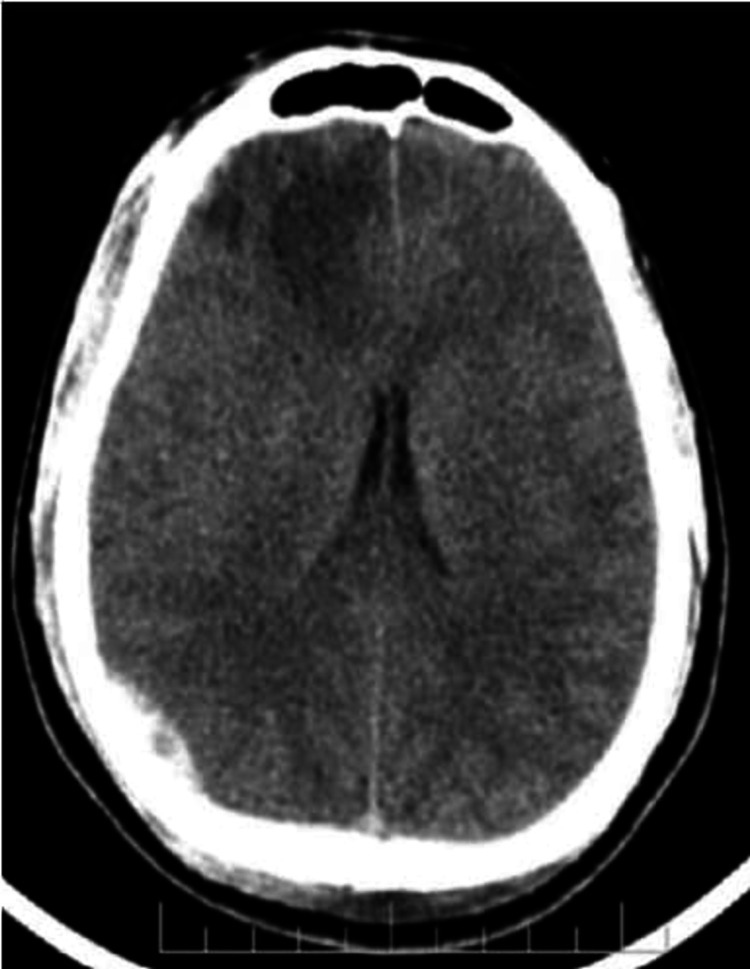
Axial NECT shows an intraparenchymal contusion in right frontal region, EDH in right parietal region with minimal mass effect compressing basal cisterns, no midline shift, subarachnoid hemorrhage noted - Rotterdam score 4 NECT: non-contrast-enhanced computed tomography; EDH: extradural hemorrhage

**Figure 11:**
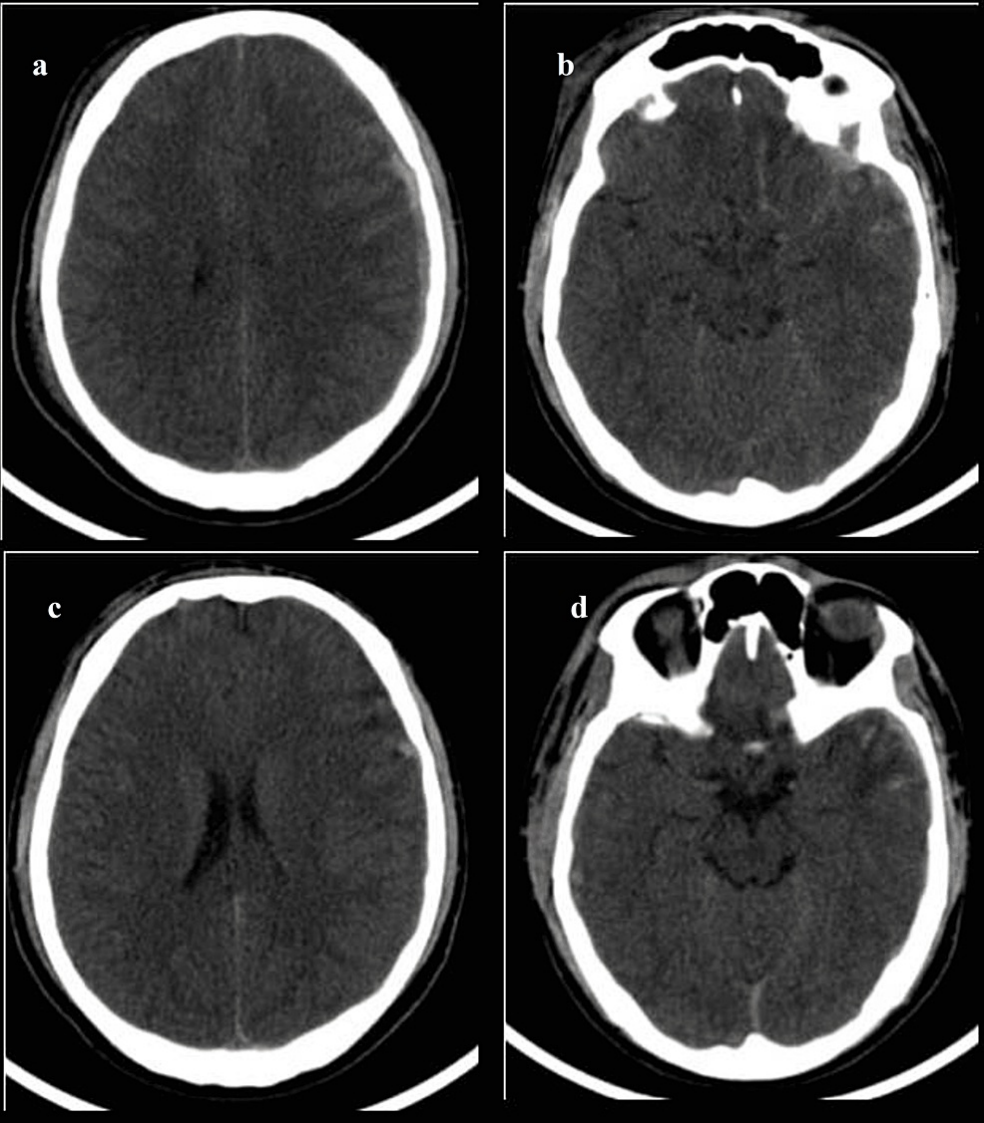
CT of a 21-year-old male patient following road traffic accident Initial admission CTs show (a) subdural hemorrhage in the left frontoparietal convexity with effacement of basal cisterns and (b) traumatic subarachnoid hemorrhage - Rotterdam score 4. Sequential CTs after 24 hours show (c and d) significant reduction in subdural hemorrhage resolution of the basal cisternal effacement, however, subarachnoid hemorrhage persisted - Rotterdam score 3.

**Figure 12:**
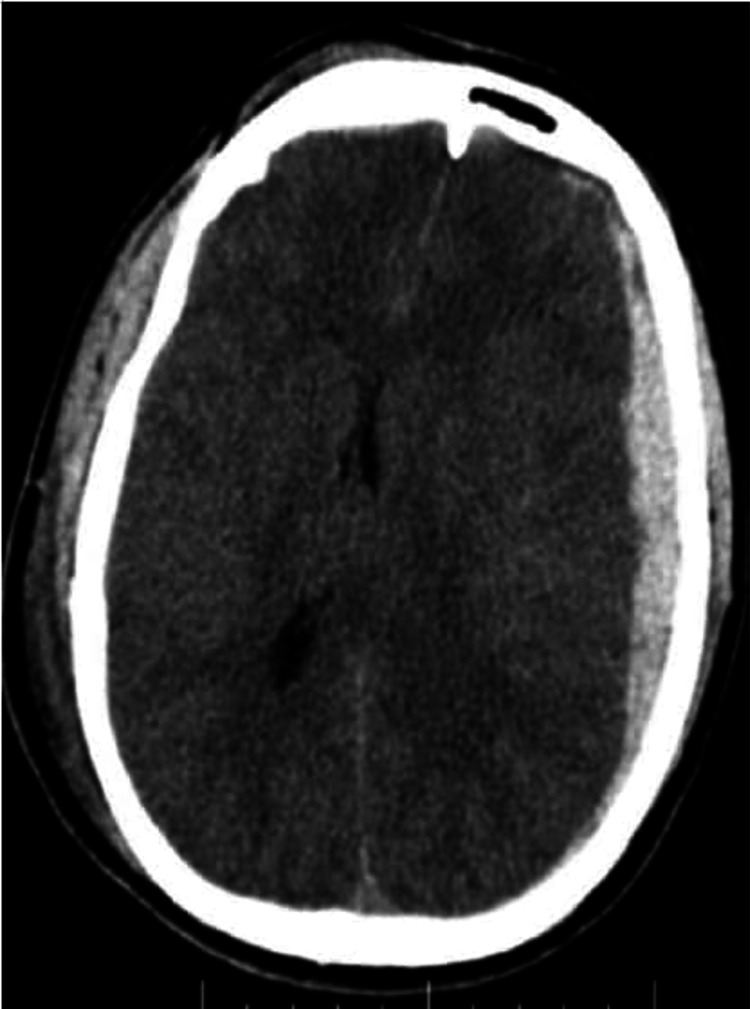
Axial NECT shows subdural hemorrhage in the left cerebral hemisphere, with mass effect compressing basal cisterns and lateral ventricles, midline shift more than 5mm, subarachnoid hemorrhage noted - Rotterdam score 5 NECT: non-contrast-enhanced computed tomography

Rotterdam score was assigned to all the cases referred for imaging in view of trauma. Baseline scores were tabulated and the change in score was noted over sequential CTs. Of those with a score of 2 (Figure 6), 29 cases (93.5%) remained unchanged over the subsequent CTs, while two (6.5%) of them worsened (Figure 7). Thirty-one cases (83.8%) of the 37 cases scored 3 (Figure 8) on the baseline CT study showed no significant change in imaging while about six (16.2%) of them did (Figure 9). Of the 13 cases with a score of 4 on the baseline CT, 10 (76.9%) of them showed signs of progression on sequential CT (Figures 10, 11). Patients with a score of 1 on the initial CT imaging were only five in number and no signs of progression were noted over the next sequential CT (Figure 5 and Table 7). Definite progression was noted with higher grades of baseline Rotterdam score, most significant being for scores 4 (76.9%) and 5 (100%), with statistically proven significance obtained on further analysis (P = 0.001) (Figure 12).

 

**Table 7:** Tabular representation of the assessment of change of Rotterdam score over sequential CT

			Changed vs unchanged	
			Changed	Unchanged
Rotterdam score	1	N (%)	0 (0%)	5 (100%)
					2	N (%)	2 (6.5%)	29 (93.5%)
	3	N (%)	6 (16.2%)	31 (83.8%)				
					4	N (%)	10 (76.9%)	3 (23.1%)
	5	N (%)	2 (100%)	0 (0%)				
					Total		N (%)	20 (22.7%)	68(77.3%)

Maximum number of sequential CTs were performed for cases presenting with a score of 3 (34 cases; 92%) with about three of them requiring up to a total of three CTs. Additionally, 27 cases (87%) with baseline score of 2 warranted a single sequential CT with two requiring up to two repeats and two requiring up to three repeat CTs. About eight cases of the total 88 required one-third cycle of CT while only two cases needed up to four CTs. The cases belonging to the latter had baseline scores of 2 (Table 8).

**Table 8:** The number of sequential CTs performed for each baseline Rotterdam CT score

Baseline Rotterdam score	Number of cases		
	CT 2	CT 3	CT 4
1	5	-	-
2	27	2	2
3	34	3	-
4	10	3	-
5	2		--

CT doses were classified into low and moderate categories of 5-10mSv and 15-25mSv, respectively. A total of 86 (97.7%) patients were exposed to low-dose CTs over the whole curse of treatment while only two cases were exposed to high cumulative radiation dose (Table 9).

**Table 9:** The number and percentage of cases being exposed to varied levels of radiation

		Frequency	Percentage
Distribution	Low (5-10mSv)	86	97.70%
	Moderate (15-25mSv)	2	2.30%
	Total	88	100%

## Discussion

CT is the single most informative diagnostic modality in the evaluation of a patient with a head injury for accurate and rapid assessment of the cranium & intracranial pathologies. In addition, CT is cost-effective and non-invasive [1-3].

The need for repeated CTs in patients with post-traumatic head injury has helped plan patient care thereby bringing about improved patient outcomes. However, different studies have been performed and varied results with contradicting results were obtained. Some stated the need for sequential CTs irrespective of clinical deterioration [4-6] while some proved the need for the same in patients with worsening clinical conditions [7-9].

CT has been used as the primary imaging modality for all cases of post-traumatic injury. Rapid clinical deterioration post-traumatic head injury is a possibility and thus decisions regarding timely effective management may be taken if an early diagnosis is known [10]. Due to its promptness, easy accessibility, cost-effectiveness, and high susceptivity to detect hemorrhage, patient outcomes and mortality are largely planned based on CT findings [11]. Defining minor versus major head injuries has been problematic.

Marshall et al. compiled various CT imaging features and put forward a classification system for organizing and grouping patients with traumatic brain injury [12]. This system allows the identification of patients at risk of deterioration from intracranial hypertension and offers the possibility of early intervention. However, SAH secondary to trauma was not considered as a factor to grade patients as part of this grading system. Also, studies have shown that the addition of SAH to a classification system may improve its ability to better stage and further plan patient management [13]. The Rotterdam criteria, hence, have proved to be a better scoring system and have been shown to be a better predictor of patient morbidity and mortality as shown in our study.

An elevated intracranial pressure (ICP) is an important factor to assess the patient’s current clinical state and a prognosticator of the probable outcome. Rotterdam CT scoring system on admission is a well-known severity assessment tool for cases of traumatic brain injury. Sonological and CT assessments of optic nerve sheath diameter (ONSD), which have been used as non-invasive indicators of raised ICP, are seen to correlate well with Rotterdam scores as well as invasive manometrically measured ICP [14-18].

This is one of the few studies on the Rotterdam score with limited available literature, especially in the Indian subcontinent. In this study, of the 88 cases analyzed a maximum number of CTs were obtained for those with a baseline Rotterdam score of 3. Of these cases, most cases did not show any change in the score or any significant progression of finding in the scans done prior to 24 hours. Cases obtaining a score of 1 and 2 did not show any change in imaging findings even after a 12- and up to 24-hour interval, thus obviating the need for a repeat CT, unless clinically indicated. However, a significant demonstration of progression on serial CTs was noted in cases with baseline scores of 4 (76.9%) and 5 (100%) with proven statistical significance (P = 0.001). On the contrary, a relatively lesser extent of progression was noted in cases with lower scores. The number of cases with baseline scores of 4 and 5 was fewer in number and we attribute this to a likely poorer prognosis and probable clinical deterioration. Surgical intervention is imperative in this sub-group of patients.

**Figure 13:**
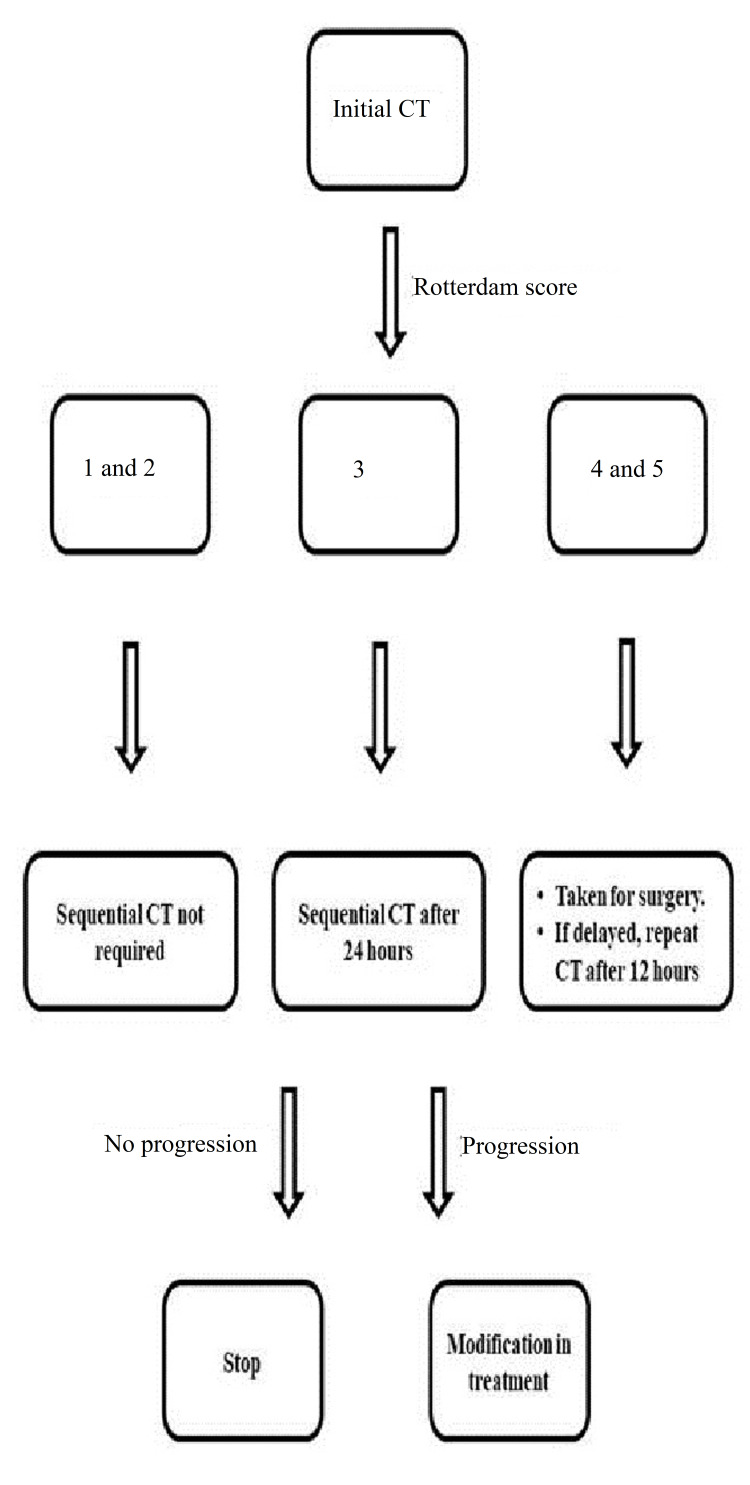
Simplified protocol for management of head trauma cases The image is created by the authors (Sachin P Shetty, Anupama Chandrappa, Sudha K Das, Kamal K Sen, and Divya V Kini) of this study.

Based on these observations, we are of the opinion that there is no additional role of sequential CT for the cases with Rotterdam score of 1 or 2 on the initial CT, unless there is clinical evidence of deterioration. Rotterdam score 3 requires a sequential CT after 24 hours and Rotterdam scores 4 and 5 need sequential CT after 12 hours if surgical intervention is delayed. Also, maximum CTs were obtained for patients with scores of 2 and 3 thus contributing to significant exposure to high radiation doses. A significant reduction in this can also be obtained by minimalizing the serial CTs for patients with lower baseline scores. To simplify this, upon analysis of the observations and results of this study, we have designed the following protocol as a simple problem-solving tool when it comes to the management of head trauma cases (Figure 13).

Although an effort has been made to design a protocol through the results obtained in our study, a few limitations were noted. This study has been performed on a small set of patients. This may be attributed to a large number of patients being referred for surgical intervention and thus being excluded from the study. Also, the Glasgow Coma Scale (GCS) scores and Rotterdam CT scores were not correlated. We included patients who had no surgical intervention after the first CT and most cases with severe traumatic brain injury were excluded. As our institute is not a primary trauma care center, input of total cases on average is lesser as compared to corresponding designated primary trauma care centers catering to this area. Thus, further validation of the study on larger and more diverse populations in varied tiers of health care institutions in order to produce a general consensus is warranted. However, as a basic management protocol in any health care institute with a provision of basic intensive care facilities, the guideline as presented by us is a simple tool to foresee and devise patient management.

## Conclusions

CT in conjunction with Rotterdam score helps in predicting any further need for a second CT, hence decreasing the unwanted radiation exposure. There is no additional role of sequential CT for the cases with Rotterdam score of 1 or 2 on the initial CT, unless there is clinical evidence of deterioration. Rotterdam score 3 needs sequential CT after 24 hours, and Rotterdam scores 4 and 5 need sequential CT after 12 hours if surgical intervention is delayed due to varied reasons. CT scan of the head incurs minimal radiation exposure of ~3.2 mSv and cumulative radiation dose according to number of sequential CT scans undergone, besides utilizing hospital manpower and machine use.

Thus, it is justifiable to conclude that non-contrast CT of the brain is a simple, inexpensive, highly effective, and safe non-invasive imaging modality that should continue to form the cornerstone of rapid diagnosis and risk stratification in traumatic brain injury.
